# Projections of non-communicable disease and health care costs among HIV-positive persons in Italy and the U.S.A.: A modelling study

**DOI:** 10.1371/journal.pone.0186638

**Published:** 2017-10-23

**Authors:** Mikaela Smit, Rachel Cassidy, Alessandro Cozzi-Lepri, Eugenia Quiros-Roldan, Enrico Girardi, Alessia Mammone, Andrea Antinori, Annalisa Saracino, Francesca Bai, Stefano Rusconi, Giacomo Magnani, Francesco Castelli, Priscilla Hsue, Antonella d’Arminio Monforte, Timothy B. Hallett

**Affiliations:** 1 Department of Infectious Disease Epidemiology, Imperial College, London, United Kingdom; 2 Department of Infection and Population Health, University College London, London, United Kingdom; 3 University Department of Infectious and Tropical Diseases, University of Brescia and Brescia Spedali Civili General Hospital, Brescia, Italy; 4 Clinical Epidemiology Unit, National Institute for Infectious Diseases 'Lazzaro Spallanzani', Rome, Italy; 5 Clinical Department, National Institute for Infectious Diseases 'Lazzaro Spallanzani', Rome, Italy; 6 Clinic of Infectious Diseases, University of Bari, Bari, Italy; 7 Department of Health Sciences, Clinic of Infectious and Tropical Diseases, ASST Santi Paolo e Carlo, University of Milan, Milan, Italy; 8 Infectious Diseases Unit, DIBIC 'Luigi Sacco', University of Milan, Milan, Italy; 9 Department of Infectious Diseases, S. Maria Nuova IRCCS Hospital, Reggio Emilia, Italy; 10 Division of Cardiology, Department of Medicine, University of California, San Francisco, San Francisco, CA, United States of America; Universita degli Studi di Roma Tor Vergata, ITALY

## Abstract

**Background:**

Country-specific forecasts of the growing non-communicable disease (NCD) burden in ageing HIV-positive patients will be key to guide future HIV policies. We provided the first national forecasts for Italy and the Unites States of America (USA) and quantified direct cost of caring for these increasingly complex patients.

**Methods and Setting:**

We adapted an individual-based model of ageing HIV-positive patients to Italy and the USA, which followed patients on HIV-treatment as they aged and developed NCDs (chronic kidney disease, diabetes, dyslipidaemia, hypertension, non-AIDS malignancies, myocardial infarctions and strokes). The models were parameterised using data on 7,469 HIV-positive patients from the Italian Cohort Naïve to Antiretrovirals Foundation Study and 3,748 commercially-insured patients in the USA and extrapolated to national level using national surveillance data.

**Results:**

The model predicted that mean age of HIV-positive patients will increase from 46 to 59 in Italy and from 49 to 58 in the USA in 2015–2035. The proportion of patients in Italy and the USA diagnosed with ≥1 NCD is estimated to increase from 64% and 71% in 2015 to 89% and 89% by 2035, respectively, driven by moderate cardiovascular disease (CVD) (hypertension and dyslipidaemia), diabetes and malignancies in both countries. NCD treatment costs as a proportion of total direct HIV costs will increase from 11% to 23% in Italy and from 40% to 56% in the USA in 2015–2035.

**Conclusions:**

HIV patient profile in Italy and the USA is shifting to older patients diagnosed with multiple co-morbidity. This will increase NCD treatment costs and require multi-disciplinary patient management.

## Introduction

Recent efforts to improve HIV care cascades have highlighted engagement levels in care globally and the challenges with starting and maintaining HIV-positive people on antiretroviral therapy (ART) [1]. With effective ART and extended life expectancy [2] new challenges are arising around the long-term management of patient relating to the increasing burden of non-communicable diseases (NCDs) [3]. Although the pathophysiological mechanisms of NCDs in the context of HIV are not fully understood, it likely involves complex interactions between traditional risk factors, including smoking, diet, and exercise, and HIV-specific risk factors, for example long-term immune activation and inflammation and toxicity related to long-term ART use [3–8].

A recent model-based study provided the first insight into the clinical implications of an ageing population in The Netherlands [9]. It showed that the mean age of HIV-positive patients is expected to increase rapidly in the coming 15 years, accompanied by a sharp increase in the burden of both NCDs and use of co-medication (in addition to ART). The study highlighted the clinical implications of this disease shift, estimating that 40% of patients will likely experience complications due to contra-indications or drug-interactions between co-medication and recommended ART by 2030 [9]. Having robust national projections for the nature and scale of NCD burden and likely economic implications for HIV care will help inform national and regional guidelines regarding the screening, monitoring and treatment of NCDs within HIV care.

In this study we adapted an individual-based model of the ageing HIV-positive population on ART [9] to Italy and the United States of America (USA) by using real-world data to provide the first projections of the changing NCD burden and direct NCD treatment costs associated with treating these increasingly complex patients in those countries in the coming two decades.

## Materials and Method

### Model

**Fig 1:**
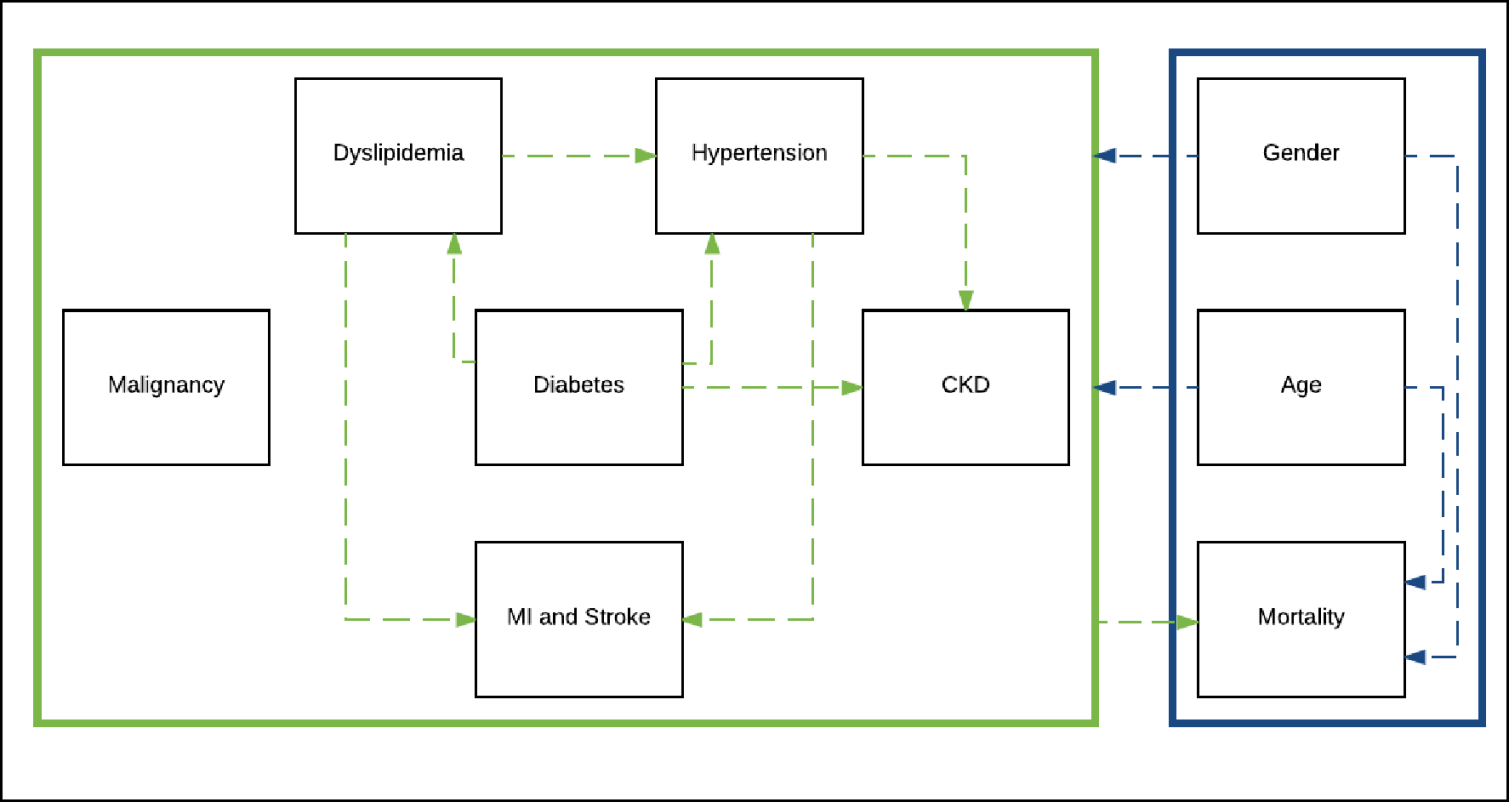
Basic schematic of the model design. The model follows patients on antiretroviral therapy from 2010 to 2035 or death. The model simulates how patients age over time and develop clinical events, including non-communicable diseases or death. The model takes into account key interactions between demographic factors (blue), e.g. how age and gender can impact risk of death, and clinical factors (green) e.g how hypertension can increase the risk of chronic kidney disease or death. *Adapted from source*: *Smit et al* [9]. *Abbreviations*: *Myocardial Infarction (MI)*, *Chronic Kidney Disease (CKD)*.

Two individual-based models of the ageing HIV-positive population were developed, one for Italy and one for the USA, by adapting an existing model [9]. Fig 1 illustrates the basic model structure, with full details of model design in the supplement (S1 File). Briefly, the model followed the ageing Italian and US HIV-positive population on ART from 1^st^ January 2010 to 31^st^ December 2035, and probabilistically simulated clinical events (hypertension, dyslipidaemia, chronic kidney disease (CKD), diabetes, ever being diagnosed with a non-AIDS defining malignancies, ever having a stroke or myocardial infraction (MIs), and mortality). We referred to hypertension and dyslipidaemia as ‘moderate’ cardiovascular disease (CVD) and ever having a MI or stroke as ‘serious CVD’. NCD risk is based on patient’s age and sex, and the propensity for one NCD to increase the risk of another NCD (Fig 1 dashed arrows), e.g. for hypertension to increase CKD risk.

Each model was first constructed at ‘cohort’ level (limited to representing only the nationally representative patient cohort for which data were available), in order to carry out-of-sample validation checks (against 2010 to 2015 data hold-outs) (Section 6 in S1 File). The model was then extrapolated to national level assuming patterns observed in the cohorts were fully representative of the national level, and by combining with national surveillance data on HIV incidence, ART coverage and number of people on ART to scale the cohort of modelled patients.

Demographic factors of HIV-positive patients on ART in 2010 (at the start of the model) were assigned probabilistically using frequency distributions from the cohorts. Rates of entry into care were based on estimated number of patients starting ART calculated using national surveillance data (Section 4 in S1 File) and assuming a conservative medium HIV-incidence scenario (i.e. a small and gradual reduction in HIV incidence up to 2035) and constant ART coverage. Results for additional HIV incidence and ART coverage scenarios can be found in the supplement (Section 7 in S1 File), including a scenario with constant HIV incidence, as reported in recent years in Italy and USA [10–12]. Disease-specific mortality rates by age and sex were taken from a large multi-cohort study, and adjusted for national mortality estimates (Section 2 in S1 File).

Existing NCDs amongst patients in 2010 (at the start of the model) were modelled using age-and-sex-specific prevalence estimates and new NCD events simulated using age-and-sex-specific incidence estimates per 1,000 person-years of follow-up from cohort data (Section 3 in S1 File). Parameters describing the propensity for one NCD to increase the risk of another NCD (Fig 1 dashed arrows) were taken from the existing model by Smit et al [9] (Section 3 in S1 File). All NCDs were defined in accordance with clinical and laboratory guidelines for diagnosis [13] (Section 3 in S1 File).

The models were used to generate projections of age structure and NCD burden for each country. All model results were based on the average of 100 model simulations.

### Data

The models were parameterised using data from HIV clinical follow-up from the Italian ICONA (Italian Cohort Naïve from Antiretrovirals) Foundation Study and from the Truven MarketScan dataset of commercially (privately) insured patients’ claims data from the USA. All patients aged ≥18 years old, infected with HIV-1 only, who were ART-naïve prior to entering the cohort were included. Table 1 provides a basic description of the patients included in the study.

**Table 1:** Demographic characteristics of the ICONA cohort and cohort of commercially-insured US HIV-positive patients in 2010 used for model parameterization. *Abbreviations: Antiretroviral therapy (ART); interquartile range (IQR); United States of America (USA)*.

	Italy (n = 1,724)	USA (n = 2,268)
**Sex**		
Male	1253 (72.7%)	1,862 (82.1%)
Female	471 (27.3%)	406 (17.9%)
**Age in 2010, years (mean)**	43.89	44.4
**Time on ART**		
<1 year	52 (3.0%)	154 (6.8%)
1–5 years	893 (51.8%)	958 (42.2%)
>5 years	779 (45.2%)	1156 (51.0%)
**Median CD4 cells/mm3 count in 2010 (IQR)**	501 (347, 699)	398 (273, 539)

The Italian model was parameterised using information from 7,469 HIV-positive patients receiving care from 1997 onwards at one of the 42 infectious disease centres across the country which contribute data to the ICONA, which was established in 1997 and whose design has been described previously [14]. A baseline population of 2,774 patients who started ART from 1st January 1997 to 31st December 2009 were used to parameterise the model, and 4,695 patients on ART who were in care from 1st January 2010 to 31st December 2015 were used to carry out out-of-sample validation.

The US model was developed using data on 3,748 commercially-insured HIV-positive patients in the USA. The data reflected a retrospective analysis of commercially-insured HIV-positive patients in the US who were drawn from a geographically representative national sample [15]. The model was parametrized using 2,268 HIV-positive individuals with record of prescribed ART from 1^st^ January 2005 to 31^st^ December 2009, with 1,480 patients from 31st December 2010 to 31st December 2014 to carry out out-of-sample validation.

Per capita direct treatment costs of HIV and NCDs were collated from insurance data in the USA and from the Brescia Local Health Agency (Brescia, Italy) database which tracks all services provides by the National Health Service [16]. Treatment was assumed to be lifelong for all NCDs, except for MIs, strokes and malignancies, were treatment was assumed to last one year. The method for cost collation for Italy has been described previously [16,17], and in the USA comparing costs in HIV-positive patients with NCDs to matched HIV-positive patients without NCDs (Section 5 in S1 File) [15].

## Results

**Fig 2:**
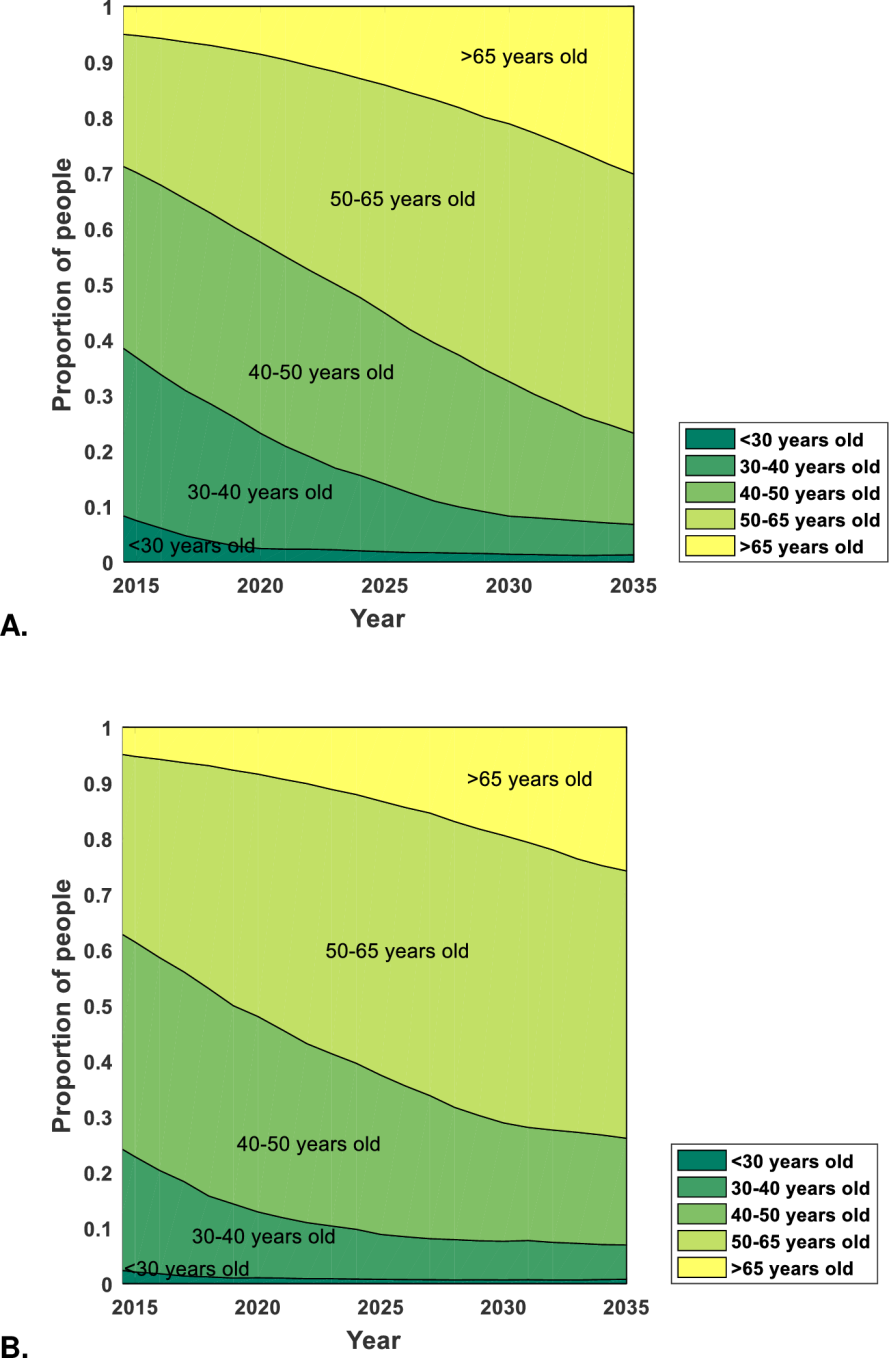
**The age distribution of HIV-positive patients on antiretroviral therapy** in **A.** Italy and **B.** the USA between 2015 and 2035.

The model predicts that mean age of HIV-positive patients will increase from 46 to 59 in Italy and from 49 to 58 in the USA (Fig 2) in 2015–2035. The proportion of patients ≥50 years old is predicted to increase from 30% and 39% in 2015 to 76% and 74% by 2035 and the proportion ≥65 years old from 5.5% to 29.3% and from 5.4% to 26.9% in Italy and the USA in 2015–2035, respectively (Fig 2). Even if mean age of new patients remained at 2010 level, mean age in 2035 would be 58 in Italy and 57 in the USA. If HIV incidence declines more rapidly or slowly, mean age by 2035 is predicted to rise to 60 and 58 in Italy and 58 and 58 2in the USA, respectively (Section 7 in S1 File).

**Fig 3:**
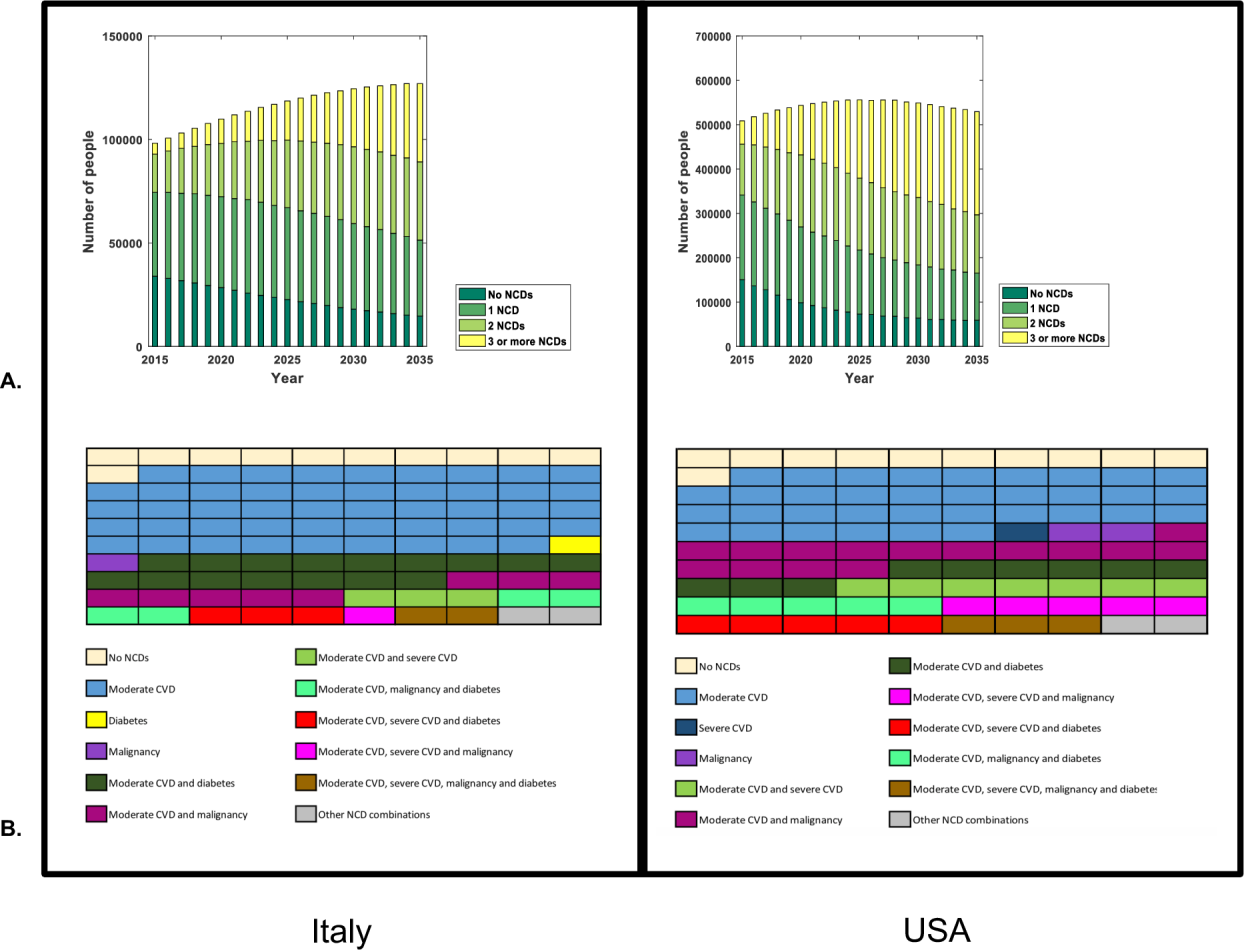
The predicted change in non-communicable disease burden. **A.** The number of HIV-positive patients on antiretroviral therapy with 0,1, 2 or 3 and more non-communicable diseases between 2015 and 2035 in Italy and USA. *Note*: *the two graphs are not on the same axis*. ***B*.** The predicted co-morbidities profiles amongst HIV-positive patients on antiretroviral therapy in 2035 based on a cross-section of 100 patients (each square represents one patient) for Italy and the USA. *Abbreviations*: *Cardiovascular disease (CVD)*, *Chronic kidney disease (CKD)*, *Moderate CVD (dyslipidemia and/or hypertension) and Severe CVD (MI or stroke); United States of America (USA)*.

In 2015, we find that the majority of patients in Italy and the USA were diagnosed with either no NCDs (35% in Italy, 29% in the USA) or 1 NCD (42% in Italy, 37% in the US) (Fig 3A). The NCD burden is expected to increase over time, with a large proportion of patients diagnosed with either 2 NCDs (31% in Italy, 25% in the US) or ≥3 NCDs (29% in Italy, 44% in the US) by 2035 (Fig 3A).

The increasing burden of NCDs will be driven by moderate CVD (hypertension, dyslipidaemia), diabetes and ever being diagnosed with a malignancy in both countries (Fig 3B). In 2015, moderate CVD contributes the greatest burden, affecting 60% and 61% of all HIV-positive patients on ART in Italy and the USA, respectively, followed by diabetes (9% and 12% respectively) and malignancies (6% and 14%). The trend is expected to persist in 2035, with the risk of being diagnosed with moderate CVD (affecting 85% of HIV-positive patients in Italy and 84% in the USA), diabetes (affecting 27% of patients in Italy and 23% of patients in the USA), or ever being diagnosed with a malignancy (affecting 16% of patients in Italy and 30% of patients in the USA) increasing.

When comparing the proportion of those with moderate (either dyslipidaemia or hypertension) and serious (ever having a MI or stroke) CVD in both countries, HIV-positive patients in the USA are expected to have double the burden of serious CVD (Fig 3B) compared to Italy by 2035 (21% in the USA versus 10% in Italy) with similar burdens of moderate CVD expected in both populations.

Differences in the demographic shift and increase in NCD burden (faster in Italy compared to the USA) appear to be driven by the differing HIV incidence. Higher HIV incidence in the USA will likely resulting in a steady addition of new, young patients to care compared to Italy where the existing population ages steadily and fewer new patients join care. Differences in serious CVD between the two settings are driven by the higher age-specific prevalence and incidence of serious CVD observed in the USA compared to Italy.

**Fig 4:**
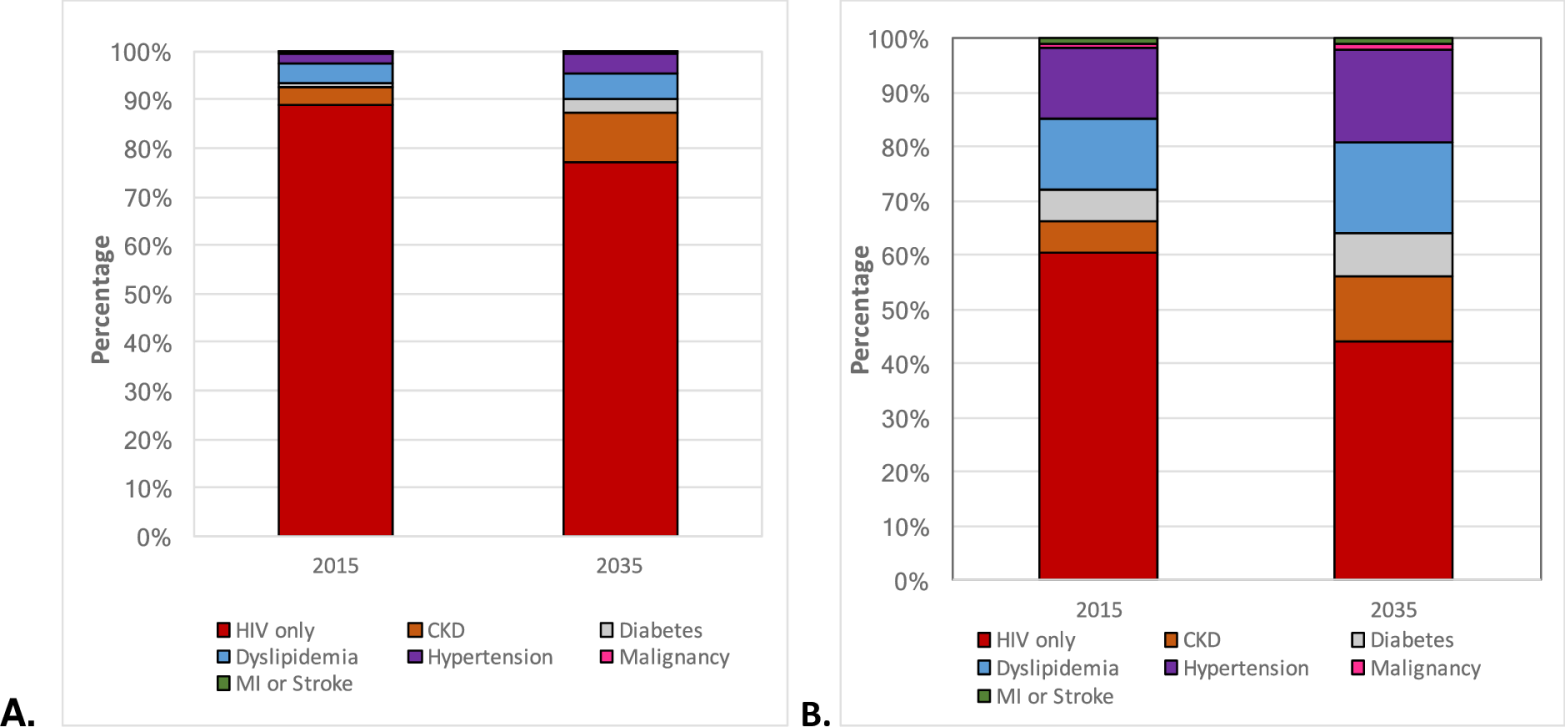
**Percentage contribution of HIV and NCD treatment costs to overall costs of care of HIV-positive patients on antiretroviral therapy in 2015 and 2035** in **A.** Italy and **B.** the USA. *Abbreviations*: *Chronic kidney disease (CKD)*, *Myocardial infraction (MI)*.

Annual direct NCD-related care costs are expected to increase between 2015 and 2035 in both countries (Fig 4). In Italy, an estimated 11.1% of total HIV direct medical care costs are associated with NCD treatment, and this is projected to increase to 22.8% by 2035 treatment. The highest contribution to overall NCD treatment relate to dyslipidaemia (3.7% in 2015 and 5.1% in 2035) and CKD (3.6% in 2015 and 10.2% in 2035), with the greatest increase relating to CKD. In the USA, direct NCD treatment costs account for 39.6% of total HIV care expenditure in 2015 treatment, and is projected to increase to 55.7% by 2035 treatment. In dollar terms, this would amount to the average cost per patient in the USA of $50,032 in 2015 ($19,812 due to NCDs) and $68,270 in 2035 ($38,050 due to NCDs), assuming no discounting. The highest contribution to overall direct NCD treatment costs are dyslipidaemia (13.3% in 2015 and 16.6% in 2035) and hypertension (12.9% in 2015 and 17.2% in 2035), and the greatest increase relates to costs of CKD (5.8% of total care cost in 2015 to 12.0% in 2035).

## Discussion

The ageing HIV-positive population in Italy and the USA will have major implications for HIV care. Our forecasts suggest that three-quarters of HIV-positive patients on ART will be aged >50 in both countries by 2035, resulting in an increased NCD burden in this population. The major drivers of NCD burdens will be hypertension, dyslipidaemia, diabetes and malignancies. These shifts will have considerable implications for direct HIV care costs, with average care costs attributable to NCD treatment expected to double in Italy and increase by 40% in the USA. Evidence based approaches on effective prevention interventions and treatment protocols will be vital to mitigate this growing burden and offset improvements in HIV care and quality of life achieved to date.

The issues of ageing with HIV are gaining momentum amongst global HIV stakeholders. UNAIDS published its “Gap report” [18] in 2014 focusing on older HIV-positive population and regional and national HIV guidelines are increasingly incorporating recommendations on the management of NCDs in HIV care [19–21]. Recently published guidelines in Italy [19], current European AIDS Clinical Society [20] and USA [21] guidelines for example provide guidance on management of NCDs in HIV-positive patients. As HIV care focus shifts from treatment of opportunistic infections to the long term prevention, screening and treatment of NCDs, particular attention needs to be given to optimal ART choice [3], management of drug-interactions between ART and NCD medication, and adherence in light of increasing polypharmacy [22]. The need for multi-disciplinary patient management with a focus on geriatric principals, personalised treatment protocols, and prevention interventions (including guidance on modifiable risk factors such as smoking, diet, exercise and other risk factors) will also be vital.

Practical application of care guidelines is an important issue; and can only become a reality when there is suitable training in place for health care providers. The evolving care requirement raises the issue of accountability; with patients receiving treatment for HIV and NCDs, the majority of care may fall on primary care physicians or HIV-specialists, although in some countries patients are already referred to general population specialists for specific NCD care. Integrated training is one possible avenue whereby HIV specialists are provided additional extensive training in geriatric medicine [23]. Yet little is known about which prevention interventions work best in the ageing HIV-positive population where there are also barriers to the prevention of NCDs such as smoking cessation [24].

The trends observed in Italy and the USA are similar to forecasts made for The Netherlands [9]. Smit et al found that by 2030 70% of HIV-positive patients on ART will be aged >50 (our forecasts show 76% for Italy and 74% for USA by 2035 and 65% for Italy and 69% for USA by 2030), and that 84% will be diagnosed with ≥NCD (our forecasts show 89% for both Italy and the USA by 2035 and 85% for Italy and 88% in the USA by 2030). As with Italy and the USA, this NCD burden will be driven mainly by CVD and diabetes. The burden of multi-morbidity (>3 NCDs) is forecasted to be higher in the USA compared to Italy and The Netherlands (28% in The Netherlands, 29% in Italy and 44% in the USA in 2030). Our current estimates of mean age are also in line with national reports, with the USA reporting that around 42% of patients are aged >50 (39% in our study) [25] and Italy reporting 29% (30% in our study) [12].

The models capture the major age-related NCDs, including their common physiological pathways. They were parameterized using large and nationally representative cohort data [15,26], and scaled to national level using national HIV surveillance data. Out-of-sample checks ensured that the model results were robust in the short-term (Section 6 in S1 File). Cost estimates of NCD care and treatment were based on proven methodology in Italy [16,17] and standard health economic method using robust insurance data from the USA.

The model is limited by the fact that it assumes homogenous national co-morbidity risk and burden in Italy and the USA. In the absence of national data on NCDs, estimates from available cohorts were assumed to directly translated to national level. As ICONA enrolls only ART-naïve patients, the average patient is likely to have less advanced disease than a typical HIV-positive patient in Italy. Although a recent study showed that recently enrolled patients were representative of Italian HIV-positive people in the European Surveillance system, ICONA may slightly over-represent men who have sex with men and underrepresent people reporting other mode of transmission, older people and those with low CD4 counts [26]. US data were generally representative of an insured US population in terms of sex, race, age and geographical distribution [15]. However, they likely represented patients with the best access to health care and not reflect patients who rely on public insurance, such as Managed or Fee-for-Service Medicaid, or those with limited or no health insurance. Extrapolation to poorer or under-served, vulnerable populations need to be made with caution; who are known to be more complex with respect to comorbidity, in part due to social or behavioral factors [27]. Further, as entry into the data requires interactions with the health care system, they may have had a small bias towards patients with greater consumption of health care resources. This may result in an underestimation of both the burden of NCDs and health care needs.

Furthermore, the model focuses on simulating HIV-positive patients in care and on ART and does not include all the NCDs that can affect these population; in particular, undiagnosed NCDs and neurocognitive or central nervous conditions which are poorly collected in HIV cohorts. This make the results of this study a conservative estimate of the true NCD burden affecting HIV-positive people in Italy and the USA in the next 20 years. The prevalence of MI, stroke and malignancy refers to ever having been diagnosed with these NCDs. The model does not account for recurrent MI, strokes or malignancies nor costs associated with recurrent events. Instead it is assumed that treatment last on average one year following the incidence of these three NCDs. As further robust age-specific data on these NCDs becomes available the model can be expanded. Estimates for annual HIV-incidence also do not take into account the lag that can occur between becoming newly infected and initiating treatment. Furthermore, the model does not account for the introduction of “test and offer” ART policies which may reduce HIV and NCD incidence [3]. Nor does the model account for migration (which may impact the burden of both HIV and NCDs, as well as the care cascade [28]) or the introduction of long-acting antiretroviral drugs (which have demonstrated good tolerability in existing trials, on drug-interactions [29]). The cost analysis for Italy are limited by the fact that cost estimates from one Italian region are extrapolated nationally, that costs for MI and strokes in Italy are based on treatment costs for a wider range of chronic CVD events excluding not MI and strokes, that frequently recovery costs in HIV-positive patients are attributed to HIV not NCDs and that compliance to NCDs treatments in Italy is likely lower than recommended protocols, (resulting in a likely underestimation of true necessary treatment costs) [16]. This lower compliance is likely due to the lack of electronic prescription system for medication for chronic diseases in Italy, meaning patients need to visit their physicians every month or two to renew prescription, affecting secondary adherence.

Finally, it is difficult to make direct comparisons between both settings due to the inherent differences in the populations, healthcare systems (mainly private in the USA and public in Italy), data sources (USA from privately insured patients, Italy ART-naïve patients) and health care systems cost differences. However, the results provide a first robust country-specific insight into the future HIV care challenges in both countries.

## Conclusions

Italian and US HIV-positive populations are ageing, increasing the NCD burden related direct treatment costs. To ensure the highest level of care is maintained for these populations, multi-disciplinary patient management and enhanced collaboration and linkage between HIV specialists, geriatric medicine and primary care must be developed. Further predictions must be made for populations where future co-morbidity estimates (and consequent care strategies) cannot be extended to, such as sub-Saharan Africa.

## Supporting information

S1 File Supplementary material. (DOCX)
