# Supplementary material for: Projections of non-communicable disease and health care costs among HIV-positive persons in Italy and the U.S.A.: A modelling study
Source: PLoS One. 2017 Oct 23;12(10):e0186638. doi: 10.1371/journal.pone.0186638 (PMC5653300; doi:10.1371/journal.pone.0186638)
Supplement: S1 File — (DOCX) [file pone.0186638.s001.docx]

**S1 File – Supplementary material**

This supplementary material provides technical details of the model designs, parameterisation, validation and additional results for sensitivity analyses. The models are an adaptation of a previous model of the ageing people living with HIV (PLHIV) in The Netherlands [1], with this supplement providing parameter values for the adapted models and outlining any major modification to the model structure. Additional details on the basic model structure can be found in the supplement by Smit and colleagues [1]. Briefly, the two models are individual-based models of an ageing HIV-positive population, one tailored to Italy and the other to the Unites States of America (USA) (Figure 1). The models follow HIV-positive patients on antiretroviral therapy (ART) from 2010, as they age, develop non-communicable diseases (NCDs), namely diabetes, hypertension, dyslipidaemia, chronic kidney disease (CKD) or experience a stroke, myocardial infraction (MI) or non-AIDS defining malignancy. Risk factors for these events are shown by the arrows in Figure 1, with the probability of events occurring evaluated at one monthly time steps in the models. These risk factors include the propensity for one NCD to increase the risk of another NCD (Figure 1), e.g. for hypertension to increase the risk of developing CKD. The individual-based models work by determining patient-level characteristics, generating cohorts, and aggregating patients against calendar time. The particulars of the model structures are outlined in detail below. Model performance was validated against 2010 to 2015 data hold-outs (see “model checks”), and predictions were made from 2015 to 2035 based on the average of 100 model runs.


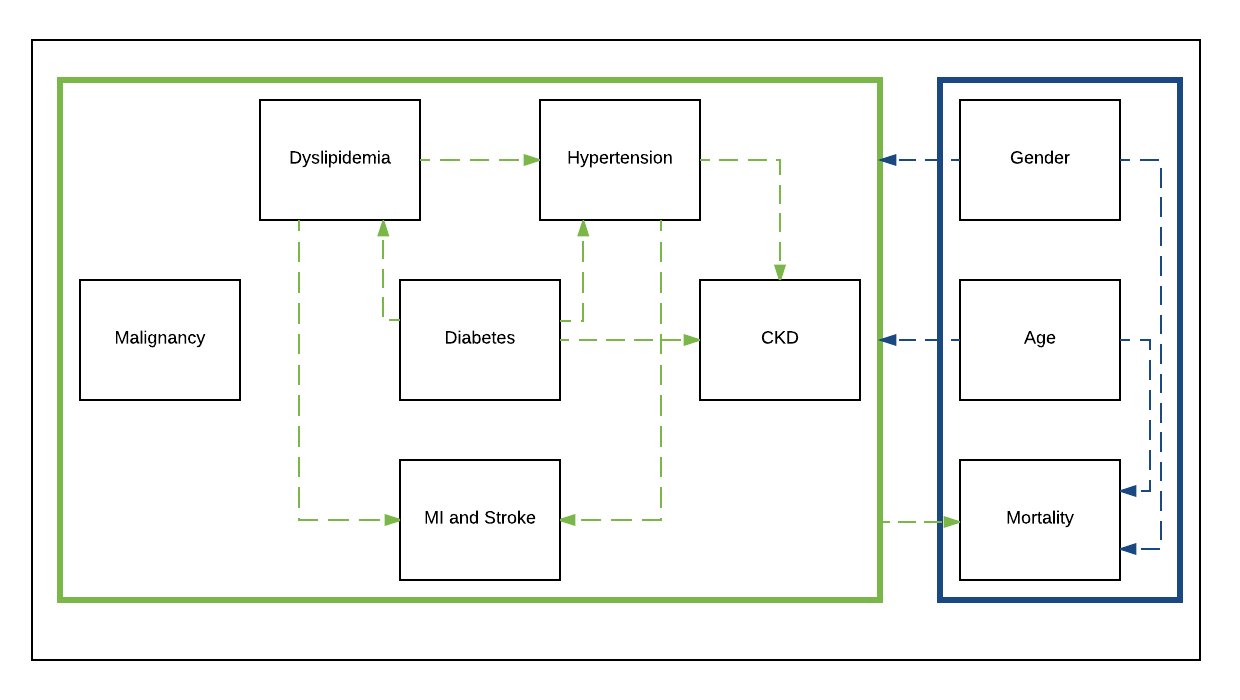


**Figure S1.** Basic schematic of the model design. The model follows patients on antiretroviral therapy from 2010 to 2035 or death. The model simulates how patients age over time and develop clinical events, including non-communicable diseases or death. The model takes into account key interactions between demographic factors (blue), e.g. how age and gender can impact risk of death, and clinical factors (green) e.g. how hypertension can increase the risk of chronic kidney disease or death.

**Section 1. Demographic Factors**

Demographic factors, specifically gender and age, were assigned probabilistically, with age assigned according to gamma distributions (which was found to best described age distribution observed in the data) (Table 1). Trends in mean age between 2015 to 2035 extrapolate from trends observed in the data prior to 2010, which show a linear increase in mean age, described as per Equation 1 where *i* stands for sex and *t* stands for calendar year. The age distributions amongst new patients joining between 2015 and 2035 are assumed to remain constant around the projected mean (Equation 2).

**Table** **S1.** Parameters describing **A.** Gender distribution**, B.** Age distribution and **C.** Annual changes in mean age, where , $\bar{Age}_{i,t} =a_{i}*{year}_{t}-b_{i}$. as per data from HIV-positive patients on ART from the ICONA study (Italy) and commercially-insured patient records (USA).

| **A. Sex Ratio** | **Italy** | | **USA** | |
| --- | --- | --- | --- | --- |
| **Male** | 0.72 | | 0.82 | |
| **Female** | 0.28 | | 0.18 | |
| **B. Age Distribution** |  |  |  |  |
| **Sex** | **Shape** | **Scale** | **Shape** | **Scale** |
| **Men** | 3.535615 | 11.43574 | 2.293965 | 19.24644 |
| **Women** | 3.253248 | 12.17016 | 2.553531 | 16.85529 |
| **C. Increase in mean age per year** |  |  |  |  |
| **Sex** | ***a_i_*** | ***b_i_*** | ***a_i_*** | ***b_i_*** |
| **Men *(i=1)*** | 0.10542 | -171.529 | 0.04571 | -49.60476 |
| **Women *(i=2)*** | 0.31656 | -597.453 | 0.39429 | -750.36193 |

Equation 1. $\bar{Age}_{i,t} =a_{i}*{year}_{t}-b_{i}$

*Equation 2.* ${Scale}_{i}=\frac{\bar{age}_{i,t}}{{shape}_{i}}$

**Section 2. Mortality**

Parameters defining cause-specific mortality rates as well as validation approach for mortality are described previously (see supplement of Smit *et al* [1]). Deaths were adapted to individual countries by adjusting the ‘background’ death rate (consisting of mortality caused due to anything other than the NCDs included in the model including for example accidental death or liver disease)[2]. This was done by accounting for differences in mean age of HIV-positive patients, as well as by fitting to annual reported number of deaths for Italy (data on deaths was not available in the commercial insurance data from the USA) as reported by ICONA.

**Section 3. NCDs**

NCDs were defined in accordance with clinical and laboratory guidelines for diagnosis [3], with full definitions used for NCDs listed in Table 2. Pre-existing NCDs amongst patients at the start of the model were assigned probabilistically using age-and-sex-specific prevalence estimates from the data (Table 3). The development of newly diagnosed NCDs are simulated based on age-and-sex-specific incidence functions while also accounting for other risk factors (such as having another NCD – see Figure 1 dashed lines between NCDs and based on parameters listed in Smit et al [1]). Age-and-sex-specific incidence functions were based on the observed incidence per 1,000 person-years of follow-up by age group and sex from data from Italian ICONA and commercial insurance data from the USA, respectively (Table 4). Functions were fitted to these incidence data to allow continuous projection of developing NCDs by age, using a least square method. Functions fitted to the data are presented in Figure 2, with their equations reported in Table 5.

**Table S2.** List of clinical and laboratory definitions used for NCDs in Italy and the USA. Abbreviations: angiotensin-converting-enzyme inhibitor (ACE inhibitor), Calcium channel blockers (CA blockers), decilitre (dL), diagnosis code (DX code), dipeptidyl peptidase inhibitor (DPP-I), Glomerular filtration rate (GFR), Glucagon-Like Peptide Receptor Agonists (GLP-RA), High-density lipoproteins (HDL), International Classification of Diseases (ICD), Italian Cohort of Patients Naïve from Antiretrovirals (ICONA), Low-density lipoprotein (LDL), micrograms (mg), millilitre (ml), millimole per litre (mmol/L), minute (min), Myocardial Infarction (MI), Chronic Kidney Disease (CKD), Non-communicable disease (NCD), sodium/glucose cotransporter 2 (SGLT2), sulfonylurea derivatives (SD), thiazolidinediones (TZD), Triglycerides (TRG), United States of America (USA).

| NCD | Definition | |
| --- | --- | --- |
|  | **ICONA (Italy)** | **Insurance data (USA)** |
| CKD | Laboratory measure:  (GFR <60 ml/min). | Laboratory measure:  (GFR <60 ml/min). |
| Diabetes | *Type I and II*  Casual glucose> 140 mg/dL.  OR  Use of at least two co-medications (insulin, SD, metformin, TZD, DPP-I, Meglitinide, GLP-RA, SGLT2-I). | *Type I and II*  Casual glucose> 140 mg/dL.  OR  Use of at least two co-medications (insulin, SD, metformin, TZD, DPP-I, Meglitinide, GLP-RA, SGLT2-I). |
| Dyslipidemia | Laboratory measure:  (Total Cholesterol>=6.2 mmol/L, LDL>=3.4 mmol/L, HDL<1 mmol/L and TRG >=2.2mmol/L) [Total Cholersterol>=239.8 mg/dL, LDL>=131.4mg/dL, HDL<38.7 mg/dL and TRG>=85.1 mg/dL].  OR  Use of at least two co-medications (statins).  *Two prescriptions (can be same medication) within a single 365-day period (primary and re-fill tied to a DX code).* | Laboratory measure:  (Total Cholesterol>=6.2 mmol/L, LDL>=3.4 mmol/L, HDL<1 mmol/L and TRG >=2.2mmol/L) [Total Cholersterol>=239.8 mg/dL, LDL>=131.4mg/dL, HDL<38.7 mg/dL and TRG>=85.1 mg/dL].  OR  Use of at least two co-medications (statins).  *Two prescriptions (can be same medication) within a single 365-day period (primary and re-fill tied to a DX code).* |
| Hypertension | Laboratory measure:  (systolic over 140 OR diastolic over 90).  OR  Use of at least two co-medications (ACE inhibitors, beta blockers, CA blockers, diuretics).  *Two prescriptions (can be same medication) within a single 365-day period (primary and re-fill tied to a DX code).* | Laboratory measure:  (systolic over 140 OR diastolic over 90).  OR  Use of at least two co-medications (ACE inhibitors, beta blockers, CA blockers, diuretics).  *Two prescriptions (can be same medication) within a single 365-day period (primary and re-fill tied to a DX code).* |
| Malignancy | Clinical diagnosis in medical record.  AND/OR  ICD code. | ICD code. |
| MI or Stroke | Clinical diagnosis in medical record.  AND/OR  ICD code. | Clinical diagnosis in medical record.  AND/OR  ICD code. |

**Table S3.** Prevalence (%) of pre-existing NCDs amongst HIV-positive patients on antiretroviral therapy initiation by gender and age as per Italian ICONA cohort data and US data on commercially insurance patients. Abbreviations: Italian Cohort of Patients Naïve from Antiretrovirals (ICONA), Myocardial Infarction (MI), Chronic Kidney Disease (CKD), United States of America (USA).

|  | **Prevalence in Italy in men (%, 95% CIs)** | **Prevalence in Italy in women (%, 95% CIs)** | **Prevalence in US in men (%, 95% CIs)** | **Prevalence in US in women (%, 95% CIs** |
| --- | --- | --- | --- | --- |
| **CKD** |  |  |  |  |
| <30 | 0.00 (0.00-0.50) | 0.00 (0.00-1.60) | 0.84 (0.09-2.74) | 1.36 (0.01-6.44) |
| 30-40 | 0.62 (0.30-1.20) | 2.25 (1.00-4.40) | 2.71 (1.17-4.14) | 3.46 (0.10-6.65) |
| 40-50 | 1.79 (1.10-2.80) | 2.19 (0.70-5.00) | 2.06 (1.11-3.09) | 4.04 (0.33-7.33) |
| 50-60 | 3.60 (2.20-5.60) | 5.88 (2.40-11.70) | 4.80 (2.91-6.78) | 5.32 (0.50-9.71) |
| ≥60 | 12.50 (8.50-17.60) | 15.00 (7.10-26.60) | 5.86 (1.67-10.22) | 11.88 (1.60-25.49) |
| **Diabetes** |  |  |  |  |
| <30 | 0.78 (0.30-1.70) | 1.28 (0.30-3.70) | 0.60 (0.24-2.85) | 0.00 (0.00-0.00) |
| 30-40 | 1.37 (0.80-2.10) | 0.56 (0.10-2.00) | 2.65 (0.45-7.20) | 3.87 (0.57-7.59) |
| 40-50 | 4.39 (3.30-5.80) | 2.63 (1.00-5.60) | 5.11 (3.75-13.11) | 8.48 (3.82-13.45) |
| 50-60 | 8.33 (6.10-11.00) | 5.04 (1.90-10.70) | 15.10 (9.97-24.01) | 17.15 (8.92-25.12) |
| ≥60 | 12.50 (8.50-17.60) | 6.67 (1.80-16.20) | 18.11 (18.91-60.31) | 41.97 (18.15-64.95) |
| **Dyslipidemia** |  |  |  |  |
| <30 | 17.28 (12.70-22.60) | 15.33 (9.70-22.50) | 5.53 (1.45-9.55) | 2.52 (0.25-19.15) |
| 30-40 | 28.37 (24.90-32.00) | 21.93 (16.70-27.90) | 11.06 (8.94-14.61) | 3.9 (1.56-8.49) |
| 40-50 | 40.13 (35.60-44.70) | 38.26 (29.40-47.80) | 19.06 (17.21-21.71) | 12.47 (7.28-18.35) |
| 50-60 | 50.82 (43.30-58.30) | 41.86 (27.00-57.90) | 27.43 (24.34-31.54) | 21.57 (14.31-29.16) |
| ≥60 | 47.62 (36.60-58.80) | 50.00 (26.00-74.00) | 29.80 (22.78-38.08) | 28.00 (9.10-46.90) |
| **Hypertension** |  |  |  |  |
| <30 | 6.61 (5.00-8.60) | 5.98 (3.30-9.80) | 3.78 (0.47-6.86) | 3.99 (0.10-15.07) |
| 30-40 | 14.62 (12.80-16.50) | 10.96 (7.90-14.70) | 7.79 (5.73-10.68) | 10.1 (4.60-15.41) |
| 40-50 | 23.30 (20.80-25.90) | 17.98 (13.20-23.60) | 10.89 (8.92-12.79) | 21.55 (14.00-28.26) |
| 50-60 | 31.82 (27.90-36.00) | 23.53 (16.20-32.20) | 19.83 (16.38-23.58) | 28.63 (20.00-37.06) |
| ≥60 | 41.96 (35.40-48.70) | 41.67 (29.10-55.10) | 25.06 (17.81-33.11) | 33.71 (12.00-54.67) |
| **Malignancy** |  |  |  |  |
| <30 | 0.91 (0.40-1.90) | 2.56 (0.90-5.50) | 1.43 (0.32-4.28) | 2.89 (0.47-12.89) |
| 30-40 | 1.44 (0.90-2.20) | 2.25 (1.00-4.40) | 4.81 (3.00-6.96) | 4.58 (1.00-8.29) |
| 40-50 | 1.97 (1.20-3.00) | 3.95 (1.80-7.40) | 6.68 (5.08-8.32) | 5.82 (2.02-9.94) |
| 50-60 | 3.41 (2.00-5.30) | 7.56 (3.50-13.90) | 10.05 (7.40-12.71) | 6.29 (2.34-12.24) |
| ≥60 | 8.04 (4.80-12.40) | 8.33 (2.80-18.40) | 20.48 (13.39-27.25) | 5.94 (1.58-21.29) |
| **MI or stroke** |  |  |  |  |
| <30 | 0.13 (0.00-0.70) | 0.00 (0.00-1.60) | 0.32 (0.05-2.82) | 0.00 (0.00-0.00) |
| 30-40 | 0.14 (0.00-0.50) | 0.28 (0.00-1.60) | 1.02 (0.28-1.99) | 0.99 (0.12-2.62) |
| 40-50 | 0.81 (0.40-1.50) | 0.88 (0.10-3.10) | 1.31 (0.76-2.20) | 1.94 (0.64-4.87) |
| 50-60 | 3.03 (1.70-4.90) | 0.84 (0.00-4.60) | 3.32 (1.50-5.19) | 4.05 (0.45-8.42) |
| ≥60 | 5.80 (3.10-9.70) | 3.33 (0.40-11.50) | 8.09 (2.34-13.41) | 12.41 (3.85-27.34) |

**Table S4.** Incidence per 1,000 person-years of newly diagnosed NCDs by gender and age group from the Italian ICONA cohort data and data on commercially insured patients in the USA. Abbreviations: Chronic Kidney Disease (CKD), Confidence Intervals (CIs), Italian Cohort of Patients Naïve from Antiretrovirals (ICONA), Myocardial Infarction (MI), Person years (PYs), United States of America (USA).

|  | Italy | | USA | |
| --- | --- | --- | --- | --- |
|  | **Incidence per 1,000 PYs in men (95% CIs)** | **Incidence per 1,000 PYs in women (95% CIs)** | **Incidence per 1,000 PYs in men (95% CIs)** | **Incidence per 1,000 PYs in women (95% CIs)** |
| CKD |  |  |  |  |
| <30 | 0.00 (0.00-0.00) | 0.00 (0.00-0.00) | 2.70 (0.30-53.40) | 0.60 (0.30-233.40) |
| 30-40 | 0.60 (0.30-1.10) | 1.00 (0.50-2.10) | 5.60 (1.20-20.40) | 3.10 (0.60-46.50) |
| 40-50 | 1.10 (0.60-2.00) | 2.50 (1.10-5.50) | 6.70 (2.10-17.80) | 9.30 (1.60-53.80) |
| 50-60 | 2.00 (0.90-4.50) | 3.50 (1.10-10.80) | 11.40 (3.60-30.80) | 13.90 (2.90-82.10) |
| ≥60 | 11.30 (6.10-21.00) | 15.30 (6.40-36.80) | 16.40 (3.70-83.50) | 26.20 (2.50-412.90) |
| Diabetes |  |  |  |  |
| <30 | 4.00 (1.70-9.70) | 1.20 (0.20-8.60) | 0.10 (0.00-47.70) | 0.00 (0.00-0.00) |
| 30-40 | 5.10 (3.30-7.90) | 0.60 (0.10-4.10) | 6.20 (1.50-21.30) | 7.70 (0.90-55.70) |
| 40-50 | 14.40 (10.00-20.70) | 7.60 (2.80-20.20) | 7.50 (2.60-18.80) | 16.40 (3.70-68.30) |
| 50-60 | 20.90 (13.30-32.80) | 22.80 (8.60-60.90) | 21.90 (8.50-48.10) | 19.70 (4.40-100.70) |
| ≥60 | 40.00 (21.50-74.40) | 16.70 (2.40-118.80) | 22.60 (5.00-100.40) | 45.50 (5.40-639.80) |
| Dyslipidemia |  |  |  |  |
| <30 | 27.90 (19.70-39.40) | 19.10 (11.50-31.60) | 22.50 (4.80-86.00) | 0.70 (0.00-236.80) |
| 30-40 | 42.60 (36.20-50.00) | 24.00 (17.40-32.90) | 40.50 (25.70-72.50) | 14.60 (3.10-67.20) |
| 40-50 | 69.70 (58.20-83.40) | 52.10 (35.20-77.10) | 73.90 (54.20-102.80) | 42.30 (14.70-107.70) |
| 50-60 | 88.90 (70.40-112.40) | 63.20 (35.00-114.20) | 97.90 (64.70-145.70) | 83.10 (32.60-194.60) |
| ≥60 | 105.80 (70.90-157.90) | 95.00 (35.70-253.30) | 84.10 (36.30-198.30) | 54.40 (11.50-545.40) |
| Hypertension |  |  |  |  |
| <30 | 4.00 (1.60-9.50) | 2.40 (0.60-9.60) | 24.40 (4.20-103.20) | 20.40 (14.60-214.40) |
| 30-40 | 7.10 (4.90-10.30) | 8.50 (5.00-14.30) | 48.20 (24.60-68.70) | 43.70 (5.30-64.70) |
| 40-50 | 21.70 (16.20-29.20) | 7.60 (2.90-20.30) | 75.30 (53.40-105.60) | 115.90 (62.00-313.10) |
| 50-60 | 20.10 (12.70-32.00) | 53.50 (26.80-107.00) | 37.50 (14.60-82.10) | 128.50 (47.10-361.20) |
| ≥60 | 52.20 (30.30-89.90) | 98.40 (36.90-262.10) | 143.20 (52.10-359.90) | 158.60 (16.80-449.70) |
| Malignancy |  |  |  |  |
| <30 | 1.60 (0.40-6.30) | 6.20 (2.60-15.00) | 5.80 (0.70-59.10) | 14.10 (1.70-260.60) |
| 30-40 | 2.00 (1.00-4.00) | 3.60 (1.60-8.10) | 14.20 (5.50-34.70) | 7.80 (1.40-55.70) |
| 40-50 | 5.60 (3.20-9.80) | 5.80 (1.90-18.00) | 16.60 (8.00-31.40) | 13.80 (3.30-62.60) |
| 50-60 | 9.90 (5.30-18.50) | 16.40 (5.30-50.90) | 15.40 (5.10-38.40) | 15.30 (4.50-100.50) |
| ≥60 | 19.40 (8.70-43.20) | 0.00 (0.00-0.00) | 68.70 (27.60-169.80) | 8.30 (0.90-350.00) |
| MI or stroke |  |  |  |  |
| <30 | 0.10 (0.00-1.00) | 0.00 (0.00-0.00) | 3.20 (0.40-0.30) | 0.00 (0.00-0.00) |
| 30-40 | 0.70 (0.40-1.20) | 0.30 (0.10-1.20) | 3.70 (0.60-49.90) | 5.30 (0.60-50.00) |
| 40-50 | 1.70 (1.00-2.80) | 0.80 (0.20-3.30) | 5.80 (1.70-52.80) | 9.20 (1.50-53.10) |
| 50-60 | 2.70 (1.40-5.50) | 1.10 (0.20-8.10) | 7.90 (2.00-71.00) | 7.20 (0.90-70.90) |
| ≥60 | 7.90 (3.80-16.60) | 5.90 (1.50-23.60) | 16.70 (3.10-442.70) | 20.00 (2.50-443.00) |

**Figure S2.** Incidence per 1,000 person-years of newly diagnosed NCDs for **A.** Italy and **B.** the USA. Data points are plotted in red (with the 95% CIs shown by the grey shaded area) with model fits plotted in blue. **NOTE**: Incidence model fits for dyslipidemia and hypertension are displayed using a different scale to the other NCDs. Abbreviations: Chronic Kidney Disease (CKD), Myocardial Infarction (MI), United States of America (USA).

1. ****
2. ****

**Table S5.** Equations for incidence of new NCDs per 1,000 person-years of follow-up as a function of age for HIV-positive patients on antiretroviral therapy for men and women separately. Functions are variations of f(x) or g(x); polynomial equations f$(x)=\sum_{i} \beta_{i-x^{i-1}}$ and exponential equations g(x)=a*exp(-βx). Abbreviations: Myocardial Infarction (MI), Chronic Kidney Disease (CKD), United States of America (USA).

|  | Italy | | | | USA | | | |
| --- | --- | --- | --- | --- | --- | --- | --- | --- |
|  | **Men** | | **Women** | | **Men** | | **Women** | |
|  | **Function Type** | **Parameters** | **Function Type** | **Parameters** | **Function Type** | **Parameters** | **Function Type** | **Parameters** |
| CKD | Exponential | a=0.00631 | Exponential | a=0.03382 | Quadratic | a₀=0.00 | Cubic | a₀=3.43325E-10 |
|  |  | β=0.10698 |  | β=0.08728 |  | a₁=0.05428 |  | a₁=2.0308E-08 |
|  |  |  |  |  |  | a₂=0.00258 |  | a₂=1.25104E-06 |
|  |  |  |  |  |  |  |  | a₃=0.00008 |
| Diabetes | Cubic | a₀=5.44400E-10 | Quadratic | a₀=1.25150E-06 | Quadratic | a₀=2.92786E-06 | Cubic | a₀=5.75205E-10 |
|  |  | a₁=3.14126E-08 | | a₁=0.00007 |  | a₁=0.00 |  | a₁=3.39852E-08 |
|  |  | a₂=0.000002 |  | a₂=0.00429 |  | a₂=0.00511 |  | a₂=2.0946E-06 |
|  |  | a₃=0.00012 |  |  |  |  |  | a₃=0.00013 |
| Dyslipidaemia | Cubic | a₀=0.00007 | Quadratic | a₀=0.00 | Quartic | a₀=2.8396E-08 | Quadratic | a₀=0.00 |
|  |  | a₁=0.00218 |  | a₁=0.40463 |  | a₁=1.14220E-06 |  | a₁=0.51582 |
|  |  | a₂=0.05561 |  | a₂=0.01373 |  | a₂=0.00005 |  | a₂=0.00725 |
|  |  | a₃=-0.00049 |  |  |  | a₃=0.00182 |  |  |
|  |  |  |  |  |  | a₄=-0.00002 |  |  |
| Hypertension | Exponential | a=1.60221 | Quartic | a₀=2.84066E-13 | Cubic | a₀=0.21693 | Cubic | a₀=-0.21028 |
|  |  | β=0.04960 |  | a₁=1.75480E-11 |  | a₃=0.00118 |  | a₁=-3.10488 |
|  |  |  |  | a₂=1.11877E-09 |  | a₁=3.23316 |  | a₂=0.19585 |
|  |  |  |  | a₃=7.27878E-08 |  | a₂=-0.10099 |  | a₃=-0.00171 |
|  |  |  |  | a₄=4.26282E-06 |  |  |  |  |
| Malignancy | Cubic | a₀=2.49482E-10 | Cubic | a₀=-0.01563 | Exponential | a=0.63009 | Quadratic | a₀=6.6947 |
|  |  | a₁=1.46585E-08 |  | a₁=-0.36579 |  | β=0.06665 |  | a₁=0.3069 |
|  |  | a₂=9.05013E-07 |  | a₂=0.02798 |  |  |  | a₂=-0.00378 |
|  |  | a₃=0.00006 |  | a₃=-0.00032 |  |  |  |  |
| MI or stroke | Exponential | a=0.06613 | Exponential | a=0.00553 | Exponential | a=0.85022 | Exponential | a=0.78656 |
|  |  | β=0.06831 |  | β=0.09955 |  | β=0.04230 |  | β=0.04592 |
|  |  |  |  |  |  |  |  |  |

**Section 4. Modelling approach and expansion to national level**

Each of the models were first constructed at the sub-national or ‘cohort’ level (limited to the patients in ICONA cohort or cohort of available commercially insured patients in the USA). This allowed us to carry out-of-sample checks (against 2010 to 2015 data hold-outs from ICONA study and commercial insurance data) on the models’ short-term projections (between 2010 and 2015) to ensure the models’ projections were robust (see “model checks”). The model was then extrapolated to national level by:

- Assuming that the sub-national trends in gender, age and risk of NCDs were directly translatable to national level
- Using national surveillance data on HIV incidence, ART coverage or number of people on ART to scale the cohort of modelled patients.

The major difference between the cohort and national model are the number of patients in care at the start of the model (in 2010) and the number of patients starting ART each year.

For the cohort model we made projections of the number of people starting ART each year between 2010-2035 by applying the same method as used in our previous model of the ageing HIV-positive population in The Netherlands (see supplement of Smit et al 2015 [1]). In summary, a compartmental model of the HIV incidence, disease progression and ART initiation was constructed to explore the different trajectories HIV incidence could take in the future. The model was solved for the force of infection by simultaneously fitting to the number of people diagnosed and starting ART each year as reported by the data assumed a minimum, medium and maximum HIV incidence scenario. The manuscript presents the results for the medium scenario with additional results listed at the bottom of this supplement (see section “Sensitivity analyses”). The model projects the number of people starting ART each year for each incidence scenario and the model assumes that the number of patients starting ART each month is constant over the year.

In order to extrapolate to the national level, we obtained information on HIV incidence, ART coverage and number of people on ART from Italy and the USA. For Italy we obtained estimates of annual number HIV incidence cases and people living with HIV (PLHIV) from UNAIDS [4] and ICONA. These data showed that the annual number of new infections have been more or less steady over the past 5 years. These incidence estimates were consolidated between the two sources to account for discrepancies, generating a mean estimate. Predictions for maximum HIV incidence assumes annual number of new HIV infections remained constant at the 2014 level, medium assumed an annual reduction of 50 cases and minimum an annual reduction of 100 infections up to 2035 (Table 6). ICONA reported that around 97,000 of people living with HIV (PLHIV) were on ART in 2015 with an ART coverage of about 88%. In order to calculate the number of PLHIV starting ART each year (Table 7) and to back-calculate an estimated number of PLHIV on ART in 2010 (at the start of the model) (Table 8) we assumed that around 88% of newly infected PLHIV started ART each year.

For the USA national extrapolation estimates of HIV incidence, number of people on ART, and ART coverage were obtained from CDC surveillance reports [5], and the CDC advanced query database [6]. Data on HIV incidence showed that number of new infections have been constant over recent years. Maximum HIV incidence was assumed to remain constant at 2015 level, medium incidence assumed to have an annual reduction of 100 cases and minimum incidence assumed to have an annual reduction of 200 cases up to 2035 (Table 6). The USA data reported that an estimated 1,2 million people were infected with HIV in 2010 with approximately 40% on ART. The same assumption and calculation approach as Italy was used to generate annual estimates of number of people starting ART annually (Table 7).

**Table S6.** Estimated incidence of HIV in Italy and the USA respectively. Source: HIV incidence estimates for Italy came from the ICONA study and UNAIDS modelling estimates [4]) and for the USA were taken from the annual HIV surveillance reports (2008-2009), the CDC advanced query database for HIV statistics (2010-2014)[5], and from CDC estimates (2015-2035)[6]. Abbreviations: Italian Cohort of Patients Naïve from Antiretrovirals (ICONA), Maximum HIV-incidence scenario (Max), Medium HIV-incidence scenario (Med), Minimum HIV-incidence scenario (Min), United States of America (USA).

|  | **Italy** | | | **USA** | | |
| --- | --- | --- | --- | --- | --- | --- |
| **Year** | **Min** | **Med** | **Max** | **Min** | **Med** | **Max** |
| **2008** | 4300 | 4300 | 4300 | 49070 | 49070 | 49070 |
| **2009** | 4200 | 4200 | 4200 | 46587 | 46587 | 46587 |
| **2010** | 4100 | 4100 | 4100 | 44940 | 44940 | 44940 |
| **2011** | 4000 | 4000 | 4000 | 43510 | 43510 | 43510 |
| **2012** | 4000 | 4000 | 4000 | 43165 | 43165 | 43165 |
| **2013** | 3850 | 3850 | 3850 | 42566 | 42566 | 42566 |
| **2014** | 3700 | 3700 | 3700 | 44073 | 44073 | 44073 |
| **2015** | 3600 | 3650 | 3700 | 43873 | 43973 | 44000 |
| **2016** | 3500 | 3600 | 3700 | 43673 | 43873 | 44000 |
| **2017** | 3400 | 3550 | 3700 | 43473 | 43773 | 44000 |
| **2018** | 3300 | 3500 | 3700 | 43273 | 43673 | 44000 |
| **2019** | 3200 | 3450 | 3700 | 43073 | 43573 | 44000 |
| **2020** | 3100 | 3400 | 3700 | 42873 | 43473 | 44000 |
| **2021** | 3000 | 3350 | 3700 | 42673 | 43373 | 44000 |
| **2022** | 2900 | 3300 | 3700 | 42473 | 43273 | 44000 |
| **2023** | 2800 | 3250 | 3700 | 42273 | 43173 | 44000 |
| **2024** | 2700 | 3200 | 3700 | 42073 | 43073 | 44000 |
| **2025** | 2600 | 3150 | 3700 | 41873 | 42973 | 44000 |
| **2026** | 2500 | 3100 | 3700 | 41673 | 42873 | 44000 |
| **2027** | 2400 | 3050 | 3700 | 41473 | 42773 | 44000 |
| **2028** | 2300 | 3000 | 3700 | 41273 | 42673 | 44000 |
| **2029** | 2200 | 2950 | 3700 | 41073 | 42573 | 44000 |
| **2030** | 2100 | 2900 | 3700 | 40873 | 42473 | 44000 |
| **2031** | 2000 | 2850 | 3700 | 40673 | 42373 | 44000 |
| **2032** | 1900 | 2800 | 3700 | 40473 | 42273 | 44000 |
| **2033** | 1800 | 2750 | 3700 | 40273 | 42173 | 44000 |
| **2034** | 1700 | 2700 | 3700 | 40073 | 42073 | 44000 |
| **2035** | 1600 | 2650 | 3700 | 39873 | 41973 | 44000 |

**Table S7.** Estimated number of HIV-positive patients initiating ART annually. Abbreviations: Antiretroviral therapy (ART), Maximum HIV-incidence scenario (Max), Medium HIV-incidence scenario (Med), Minimum HIV-incidence scenario (Min), United States of America (USA).

|  | **Italy** | | | **USA** | | |
| --- | --- | --- | --- | --- | --- | --- |
| **Year** | **Min** | **Med** | **Max** | **Min** | **Med** | **Max** |
| **2008** | 3784 | 3784 | 3784 | 20119 | 20119 | 20119 |
| **2009** | 3696 | 3696 | 3696 | 19101 | 19101 | 19101 |
| **2010** | 3608 | 3608 | 3608 | 18425 | 18425 | 18425 |
| **2011** | 3520 | 3520 | 3520 | 17839 | 17839 | 17839 |
| **2012** | 3520 | 3520 | 3520 | 17698 | 17698 | 17698 |
| **2013** | 3388 | 3388 | 3388 | 17452 | 17452 | 17452 |
| **2014** | 3256 | 3256 | 3256 | 18070 | 18070 | 18070 |
| **2015** | 3168 | 3212 | 3256 | 17988 | 18029 | 18040 |
| **2016** | 3080 | 3168 | 3256 | 17906 | 17988 | 18040 |
| **2017** | 2992 | 3124 | 3256 | 17824 | 17947 | 18040 |
| **2018** | 2904 | 3080 | 3256 | 17742 | 17906 | 18040 |
| **2019** | 2816 | 3036 | 3256 | 17660 | 17865 | 18040 |
| **2020** | 2728 | 2992 | 3256 | 17578 | 17824 | 18040 |
| **2021** | 2640 | 2948 | 3256 | 17496 | 17783 | 18040 |
| **2022** | 2552 | 2904 | 3256 | 17414 | 17742 | 18040 |
| **2023** | 2464 | 2860 | 3256 | 17332 | 17701 | 18040 |
| **2024** | 2376 | 2816 | 3256 | 17250 | 17660 | 18040 |
| **2025** | 2288 | 2772 | 3256 | 17168 | 17619 | 18040 |
| **2026** | 2200 | 2728 | 3256 | 17086 | 17578 | 18040 |
| **2027** | 2112 | 2684 | 3256 | 17004 | 17537 | 18040 |
| **2028** | 2024 | 2640 | 3256 | 16922 | 17496 | 18040 |
| **2029** | 1936 | 2596 | 3256 | 16840 | 17455 | 18040 |
| **2030** | 1848 | 2552 | 3256 | 16758 | 17414 | 18040 |
| **2031** | 1760 | 2508 | 3256 | 16676 | 17373 | 18040 |
| **2032** | 1672 | 2464 | 3256 | 16594 | 17332 | 18040 |
| **2033** | 1584 | 2420 | 3256 | 16512 | 17291 | 18040 |
| **2034** | 1496 | 2376 | 3256 | 16430 | 17250 | 18040 |
| **2035** | 1408 | 2332 | 3256 | 16348 | 17209 | 18040 |

**Table S8.** Estimated number HIV-positive people receiving ART in Italy and the USA. Abbreviations: Antiretroviral therapy (ART), Maximum HIV-incidence scenario (Max), Medium HIV-incidence scenario (Med), Minimum HIV-incidence scenario (Min), United States of America (USA).

|  | **Italy** | **USA** |
| --- | --- | --- |
| **Year** | **Number on ART** | **Number on ART** |
| **2010** | 80104 | 444000 |
| **2011** | 83624 | 461839 |
| **2012** | 87144 | 479537 |
| **2013** | 90532 | 496989 |
| **2014** | 93788 | 515059 |
| **2015** | 97000 | 533088 |

**Section 5. Cost analysis**

Per capita treatment costs of NCDs and HIV amongst HIV-positive patients in the USA and Italy are listed in Table 9 and disease and treatment classification used are listed in Table 10. In the USA, direct annual per capita costs per NCD were estimated by calculating incremental costs by comparing estimated consumption of pharmacy, inpatient, and outpatient healthcare resources in HIV-positive patients with the NCDs to costs for matched HIV-positive patients without the NCD. Hospitalisation costs, inclusive of procedures, hospital stay, durable medical goods and pharmacy costs (inclusive of prescription medications and excluding medications to treat HIV) were used to estimate annual, per capita costs. The method for cost collation in Italy has been previously described [7,8]. and include pharmacy (ART and all drugs reimbursed by Sanitary National System), in- and out-patient costs (including laboratory and clinical imaging) (supplement). The direct medical costs for CVD in Italy include a wider range of chronic CVD events (supplement) but not MI and strokes, and are assumed to be representative of treatment for MI and strokes. (see Table 10).

**Table S9.** Annual per capita treatment cost for HIV and NCD treatment amongst HIV-positive patients in Italy and the USA. Details of cost breakdown are available for the USA but only for HIV in Italy. Abbreviations: Cardiovascular disease (CVD), Chronic Kidney Disease (CKD), United States of America (USA).

*CVD costs in Italy include a number of cardio-cerebrovascular diseases including heart disease, cardiac insufficiency and cadriovasculopathies (see Table 10) whereas in the US the costs are restricted to stroke and Myocardial infraction. Source: For Italian cost data [7].

|  | Details | Costs Italy | Details | Costs USA |
| --- | --- | --- | --- | --- |
| HIV | Inpatient admissions | € 1262 | Inpatient admissions | $2,502 |
|  | Outpatient services | € 1544 | Outpatient services | $3,806 |
|  | ART | € 8612 | Prescription Medication (non-ART) | $2,627 |
|  |  |  | ART | $21,285 |
|  | **Total** | **€ 11,589** | **Total** | **$30,220** |
| CKD |  |  | Inpatient admissions | $12,500 |
|  |  |  | Outpatient services | $18,624 |
|  |  |  | Prescription Medication (non-ART) | $5,544 |
|  | **Total** | **€ 13,665** | **Total** | **$36,668** |
| Diabetes |  |  | Inpatient admissions | $8,124 |
|  |  |  | Outpatient services | $10,788 |
|  |  |  | Prescription Medication (non-ART) | $5,580 |
|  | **Total** | **€ 1,701** | **Total** | **$24,492** |
| Dyslipidaemia |  |  | Inpatient admissions | $9,056 |
|  |  |  | Outpatient services | $4,846 |
|  |  |  | Prescription Medication (non-ART) | $3,674 |
|  | **Total** | **€ 1,003** | **Total** | **$17,576** |
| Hypertension |  |  | Inpatient admissions | $8,544 |
|  |  |  | Outpatient services | $4,572 |
|  |  |  | Prescription Medication (non-ART) | $4,716 |
|  | **Total** | **€ 1,184** | **Total** | **$ 17,832** |
| Malignancy |  |  | Inpatient admissions | $13,004 |
|  |  |  | Outpatient services | $18,577 |
|  |  |  | Prescription Medication (non-ART) | $5,573 |
|  | **Total** | **€ 7,558** | **Total** | **$37,154** |
| CVD* |  |  | Inpatient admissions | $24,888 |
|  |  |  | Outpatient services | $17,004 |
|  |  |  | Prescription Medication (non-ART) | $4,788 |
|  | **Total** | **€ 3,699** | **Total** | **$46,680** |

**Table S10.** Methodology used to define NCDs for cost analysis. Abbreviations: Anatomic and Therapeutic Chemical Classification (ATC); Daily Defined Doses (DDD); Diagnosis Related Groups (DRGs); International Classification of Disease 9^th^ Revision, Clinical Modification (ICD-9-CM).

*the ATC/DDD codes were used for Italy. In the USA codes for medications are based on US NDC (national drug codes), and costs are associated with diagnoses rather than with specific medications, due to the high number of possible codes (for example, for statin, each brand, package, strength, dosing, etc. has its own 11-digit code). Instead pharmacy, inpatient and outpatient costs are associated with a diagnosis for a claim. Incremental cost difference with diagnosis is estimated for costs.

| Disease | Hospital Discharge Diagnoses  (DRG-ICD9CM) | Drug Prescription  (ATC/DDD/NDC)* | Ambulatory  care | Registry Residential care or Registry psychiatric facility  (SVM code)  (Italy only) | Free access for care service  (Italy only) |
| --- | --- | --- | --- | --- | --- |
| Cardiovascular diseases (Italy) | 394 395, 396, 397, 402, 403, 404, 414, 424, 426, 427, 429.4, 745,746, V42.2; V43.3; V45.0, 416; 417, 428.0; 428.1; 428.9, 416*, 417, 433, 434, 440, 441.2, 441.4, 441.7, 441.9, 442, 444, 447.0, 447.1, 447.6, 452, 453; 459.1, 557.1, 747, V43.4, 443.1 | C01, C02, C03, C07, C08, C09, BO1  (DDD for the year ≥ 70%) |  | Cardiovascular disease diagnosed at admission (moderate to severe) | Heart disease  Cardiovasculopathies |
| MIs and Stroke (USA) | 410, 411.0, 412, 434 |  |  |  |  |
| Diabetes | 250 or DRG 294 and 295 | A10A (DDD≥10%), or A10B (DDD≥30%) |  | Diabetes diagnosed at admission | Diabetes |
| Dyslipidemia | 272.0, 272.2, 272.4, 272.9 | C10 (DDD≥70%) |  | Dyslipidemia diagnosed at admission | Dyslipidemia |
| Chronic kidney failure | 585, V56 or DRG 316 and 317 |  | Dialysis service | Chronic kidney failure diagnosed at admission (moderate or severe) | Chronic kidney failure |
| Hypertension | 401, 405, DRG 134 FOR USA: also  402 | C01, C02, C03, C07, C08, C09, BO1  (DDD for the year ≥ 70%) |  |  |  |
| Malignancies | 140-208; V10, V58.1 | L01 | Oncology services chemo- and radiotherapy |  | Several cancer types |

**Section 6. Model checks**

A large number of model checks were carried out using an out-of-sample approach. That is data from 2010-2015 was ‘held back’ and not used for model parameterisation and compared to model projection between 2010 to 2015. Model checks were carried out for the ‘cohort’ models (models constructed to represent the cohort of patients in ICONA and cohort of patients available in the insurance data for the USA). These checks were carried out to ensure the model design and structure was robust prior to extrapolating to the national level. The results of these model checks show that the model consistently generates output of the right order of magnitude, leading to the conclusion that the model provides projections of the right direction. Individual discrepancies are discussed in details below.

**Section 6.1. Number of people starting ART and in care**

Results of the model output on the number of people starting ART and in care was compared to out-of-sample data from the Italian ICONA study and the USA-based insurance data (Figure 3). The discrepancies observed in this comparison including the under-estimation of the number of individuals starting ART between 2010 and 2015 in the Italian ICONA cohort model (shown in Figure 3A). This can be explained in part by ICONA experiencing a surge in recruitment post-2010 which the deterministic model is unable to account for. On the other hand, in the USA cohort model the output consistently over-estimated the number of individuals receiving ART between 2010 and 2014 (shown in Figure 3D). This can partially be explained by the high number of individuals switching insurance providers, resulting in a large of lost to follow up. These discrepancies disappear in the national model.

**Figure S3.** Number of patients starting ART in **A.** Italy and **B.** the USA and number of people in care in **C.** Italy and **D.** the USA according to data and model output. Abbreviations: Antiretroviral therapy (ART), United States of America (USA).

**A.
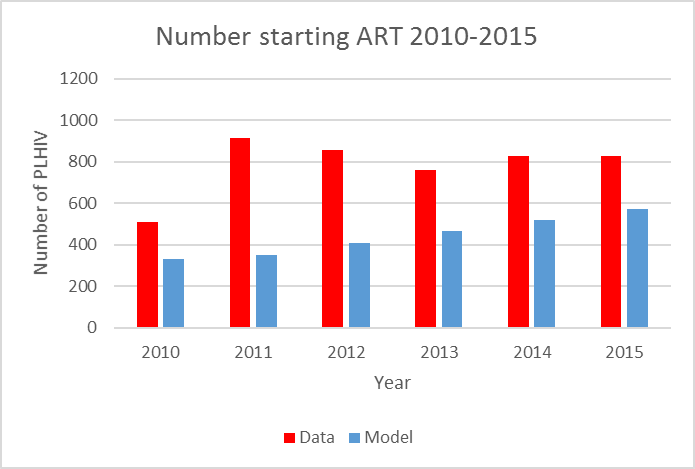
 B.
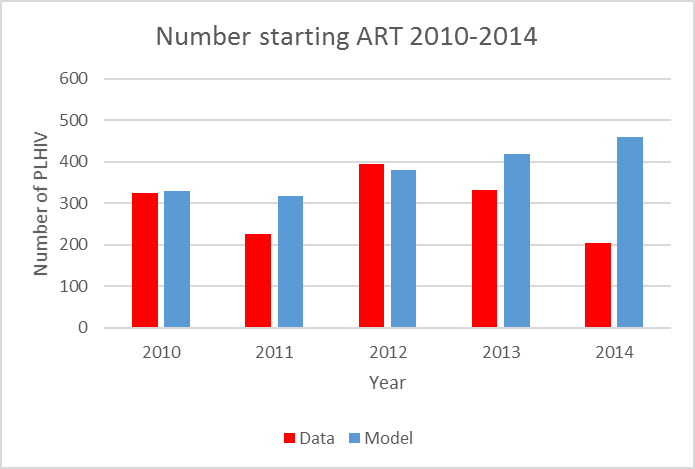
**

**C.
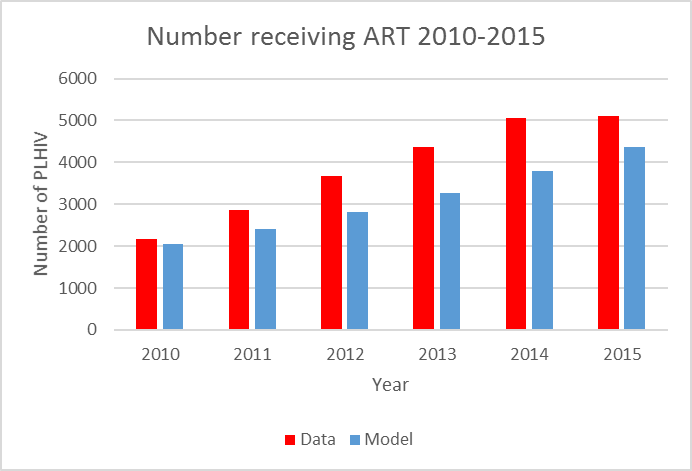
 D.
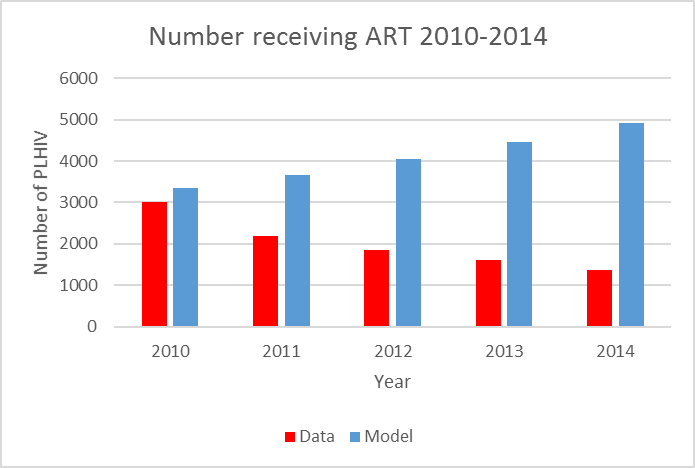
**

**Section 6.2. Mean age**

The mean age amongst patient in care was compared between model output and data from the ICONA study and USA-based insurance provider. The results show that the model is able to recreate changes in mean age in the patient population robustly (Figure 4).

**Figure S4.** Mean age amongst HIV-positive patients on ART in **A.** Italy and **B.** the USA according to data and model output. Abbreviations: Antiretroviral therapy (ART), United States of America (USA).

**A.
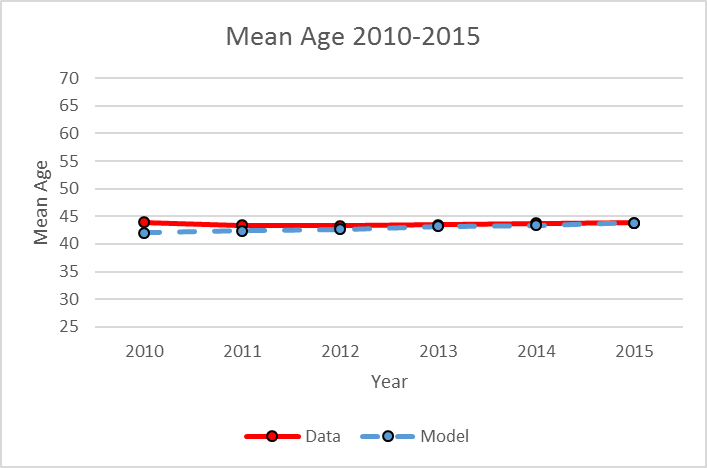
 B.
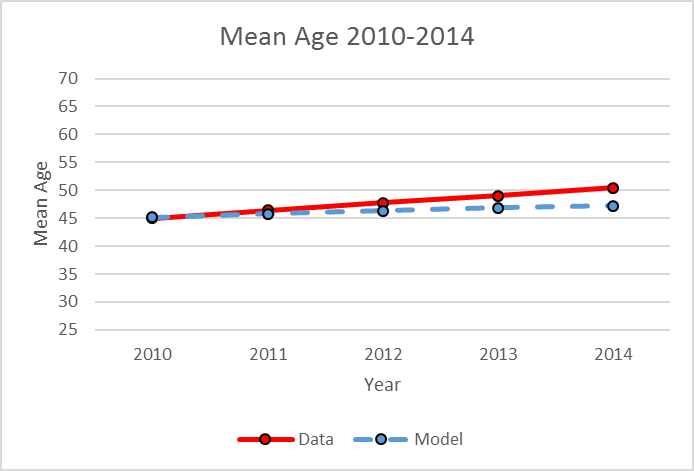
**

**Section 6.3. New NCD diagnoses**

The model simulated the development of newly diagnosed NCD through a combination of demographic factors (age and sex) and medical factors, via a system of common causal pathway with parameter values coming from different sources. In order to check the robustness of this approach, the number of people diagnosed with NCDs between 2010 and 2015 was compared between out-of-sample data and the model output. The results are presented in Figure 5 and show that the model consistently generates output of the right order of magnitude.

**Figure S5.** Annual number of new NCDs developed by HIV-positive patients on ART according to data and model for **A.** Italy and **B.** the USA. Abbreviations: Antiretroviral therapy (ART), Myocardial Infarction (MI), Chronic Kidney Disease (CKD), United States of America (USA).

**A.**

**B.**

**Section 7. Sensitivity analyses**

The below show the results with the minimum and maximum HIV incidence scenarios.

1. **B.**

**C. D.**

**Figure S6.** Projected age distribution of HIV-positive patients on antiretroviral therapy between 2015 and 2035 for **A.** Italy assuming minimum HIV-incidence, **B.** Italy assuming maximum HIV-incidence, **C.** USA assuming minimum HIV-incidence, and **D.** USA assuming maximum HIV-incidence.

1.
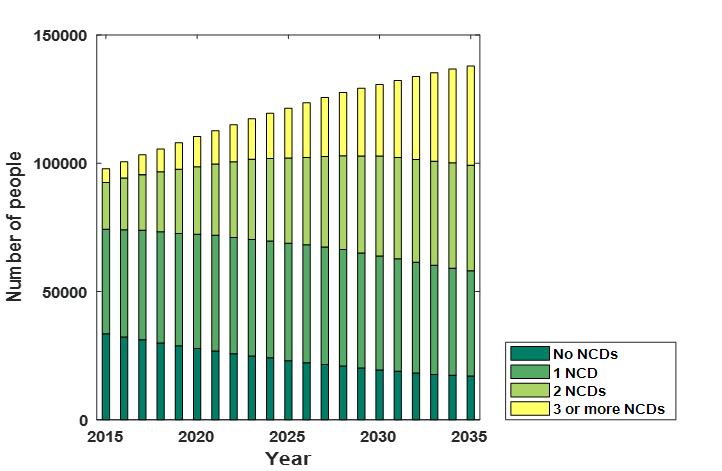
**
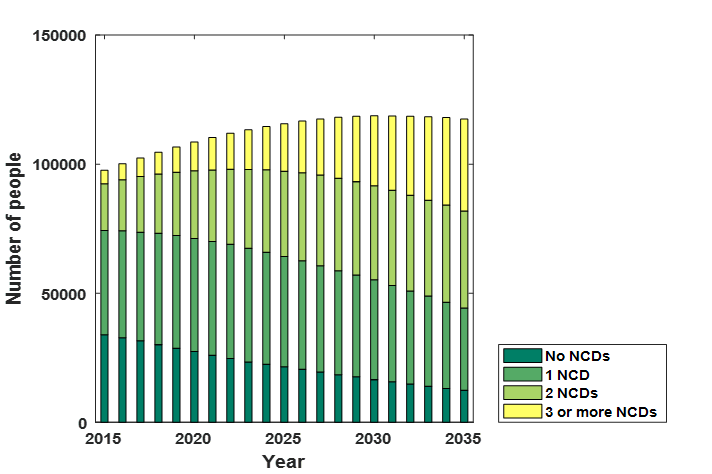
**  **B.**

**C. D.**

**Figure S7.** The projected burden of non-communicable diseases amongst HIV-positive patients on antiretroviral therapy in **A.** Italy assuming minimum HIV-incidence, **B.** Italy assuming maximum HIV-incidence, **C.** USA assuming minimum HIV-incidence and **D.** USA assuming maximum HIV-incidence.

The below shows results for additional ART coverage scenario, including one where ART coverage increases to 90:90:90 target by 2035 and one where ART coverage in Italy is 10% lower than the 88% currently assumed (Table 11).

**Table S11.** Sensitivity analyses varying ART coverage and outcomes by 2035.

| Projection in 2035 | Baseline Italy* | ART coverage reduced by 10% in Italy | Baseline USA* | ART coverage reaches 90:90:90 by 2035 in USA |
| --- | --- | --- | --- | --- |
| Mean age | 59 | 59 | 58 | 56 |
| Proportion with ≥  1 NCD | 89% | 89% | 89% | 85% |
| Proportion with ≥  3 NCD | 29% | 30% | 44% | 38% |

**Baseline defined as HIV incidence that remains stable at 2015 levels up until 2035 and where ART coverage increases steadily to 90:90:90 targets.*

**References**

1. Smit M, Brinkman K, Geerlings S, et al. Future challenges for clinical care of an ageing population infected with HIV: a modelling study. Lancet Infect. Dis. **2015**; 15:810–818.

2. Data Collection on Adverse Events of Anti-HIV drugs (D:A:D) Study Group, Smith C, Sabin CA, et al. Factors associated with specific causes of death amongst HIV-positive individuals in the D:A:D Study. AIDS Lond. Engl. **2010**; 24:1537–1548.

3. European AIDS Clinical Society. Guidelines 2013. **2013**;

4. UNAIDS. Number of people living with HIV. 2015. Available at: http://aidsinfo.unaids.org/. Accessed 17 July 2016.

5. CDC. HIV Surveillance Report, 2014. 2015. Available at: http://www.cdc.gov/hiv/pdf/library/reports/surveillance/cdc-hiv-surveillance-report-us.pdf.

6. CDC. CDC Advanced Query Database. Available at: http://www.cdc.gov/surveillancepractice/data.html.

7. Quiros-Roldan E, Magoni M, Raffetti E, et al. The burden of chronic diseases and cost-of-care in subjects with HIV infection in a Health District of Northern Italy over a 12-year period compared to that of the general population. BMC Public Health **2016**; 16:1146.

8. Magoni M, Scarcella C, Vassallo F, et al. The evolving burden of HIV infection compared with other chronic diseases in northern Italy. HIV Med. **2011**; 12:129–137.
